# Dangua Fang induces anti-glucolipid metabolism disorder effects similar to those of direct NFIL3 inhibition

**DOI:** 10.3389/fmicb.2025.1557345

**Published:** 2025-03-19

**Authors:** Zhuang Han, Linxi Jin, Zhita Wang, Liuqing Yang, Liang Li, Yi Ruan, Qiwei Chen, Shuhong Yao, Weidong He, Xianpei Heng

**Affiliations:** ^1^Department of Endocrinology, People’s Hospital Affiliated to Fujian University of Traditional Chinese Medicine, Fuzhou, China; ^2^First Clinical Medical College, Fujian University of Traditional Chinese Medicine, Fuzhou, China; ^3^Department of Geriatrics, People’s Hospital Affiliated to Fujian University of Traditional Chinese Medicine, Fuzhou, China

**Keywords:** Dangua Fang (DGF), nuclear factor, interleukin-3 regulated (NFIL3), glucolipid metabolism disorder (GLMD), gut microbiota, lipid metabolism

## Abstract

**Background:**

Dangua Fang (DGF) is a traditional Chinese herbal formula widely used to regulate glucolipid metabolism. Nuclear factor, interleukin-3 regulated (NFIL3) plays a regulatory role in intestinal fat absorption and energy metabolism. Gut microbiota can modulate NFIL3 expression and affect host metabolism.

**Purpose:**

This study aimed to investigate the effects of DGF or NFIL3 inhibition on the gut microbiota and their metabolites in mice with glucolipid metabolism disorder (GLMD) and explore the relationship between DGF anti-GLMD effects and those of direct NFIL3 inhibition.

**Methods:**

A GLMD mouse model was established by induction with a high-glucose and high-fat diet. The mice were divided into the control group (CG), model group (MG), DGF group (DFG), DGF + siRNA group (DFSG), and siRNA group (SG). The mice were administered sterile water, DGF, and/or intraperitoneal injections of siRNA-NFIL3 or normal saline for 15 weeks, following which glucolipid metabolic indicators, NFIL3 levels, and histopathological alterations in the liver and small intestinal tissues were evaluated. Additionally, the gut microbiota and differential metabolites were analysed, and linear regression analysis was conducted between gut microbial species and metabolic indicators to assess the role of the gut microbiota in metabolic regulation.

**Results:**

Significant differences were observed between the CG and MG groups for various indicators. Compared with that in the MG group, the GLMD in the DFG, DFSG, and SG groups was significantly improved, and the pathological morphology of the liver and small intestine was altered. The NFIL3 mRNA and protein expression levels in the serum, liver, and small intestine were significantly decreased. The relative abundance of Bacteroidota decreased, whereas that of Firmicutes increased, and changes in the gut microbiota significantly correlated with serum total cholesterol (TC), triglyceride (TG), and free fatty acid (FFA) levels. Moreover, lipid metabolism-related pathways were significantly altered in all three intervention groups.

**Conclusion:**

DGF reduced NFIL3 expression in GLMD mice, regulated the gut microbiota and their metabolites, and altered lipid metabolism-related pathways, with anti-GLMD effects similar to those of direct NFIL3 inhibition.

## 1 Introduction

The key pathological feature of glucolipid metabolism disorder (GLMD) is energy surplus, with excessive intestinal fat absorption being a major contributing factor (Zhang et al., 2023; Niu et al., 2020). Increased fat absorption in the intestines alters the lipid environment, thereby promoting energy storage in the body, leading to obesity, fatty liver disease, insulin resistance, and metabolic inflammation (Wit et al., 2022). Nuclear factor, interleukin-3 regulated (NFIL3), also known as E4BP4, is a central regulator of intestinal fat absorption (Wang et al., 2017). In individuals with obesity, NFIL3 mRNA levels are positively correlated with body mass index (Wu et al., 2009). Animal experiments have demonstrated that a high-fat diet (HFD) can upregulate NFIL3 expression in mice (Sun et al., 2024). NFIL3 is highly expressed in the liver, and knocking out the NFIL3 gene enhances insulin sensitivity, reduces fat accumulation, decreases the expression of inflammation-related genes, and mitigates obesity and other HFD-induced metabolic problems (Wang et al., 2023; Zhao et al., 2021). Moreover, NFIL3 induces lipid droplet formation in mouse hepatocytes by regulating lipid droplet-associated proteins (Wang et al., 2023). Therefore, inhibiting NFIL3 reduces the expression of genes related to lipid droplet association and fat uptake in hepatocytes, decelerates *de novo* lipogenesis, and alleviates HFD-induced hepatic steatosis and liver injury (Yang et al., 2020; Matsumura et al., 2021).

The gut microbiota play a crucial role in regulating energy metabolism in the body by aiding in food decomposition, affecting the absorption of long-chain fatty acids by intestinal epithelial cells, and modulating adipose tissues function as well as inflammatory responses (Brown et al., 2023; Fan and Pedersen, 2021). Additionally, the gut microbiota regulates lipid uptake and storage via NFIL3, thus affecting body composition (Wang et al., 2017). Intestinal microbiota members that produce flagellin and lipopolysaccharides modulate the circadian rhythm of NFIL3 through innate lymphoid cells 3 and activator of transcription 3, thereby influencing the diurnal fluctuations in fat metabolism pathways and affecting fat absorption and storage (Wang et al., 2017; Kubo, 2020). However, existing studies have only focused on the effects of the gut microbiota on NFIL3, and no research has confirmed a regulatory role of NFIL3 on the gut microbiota.

Dangua Fang (DGF) is a traditional Chinese herbal formula, which is derived from the combination of two classical prescriptions, Siwu Tang and Gualou Xiebai Banxia Tang, used for regulating glucolipid metabolism. It has been approved by the Fujian Provincial Food and Drug Administration and widely used in clinical settings. Animal studies have revealed that DGF can effectively reduce body weight, lower blood glucose and lipid levels, decrease peri-testicular and peri-renal fat content, increase liver/kidney and liver/spleen ratios, and inhibit arterial plaque formation (Xianpei et al., 2023, 2024; Heng et al., 2022, 2023). A three-year randomised controlled clinical trial demonstrated that DGF could effectively improve glucolipid metabolism, reduce insulin dosage in patients with diabetes, lower the ultrasound score of lower-extremity atherosclerosis, improve left ventricular diastolic function, and reduce the risk of new-onset coronary heart disease and all-cause mortality (Heng et al., 2019).

**Figure 1:**
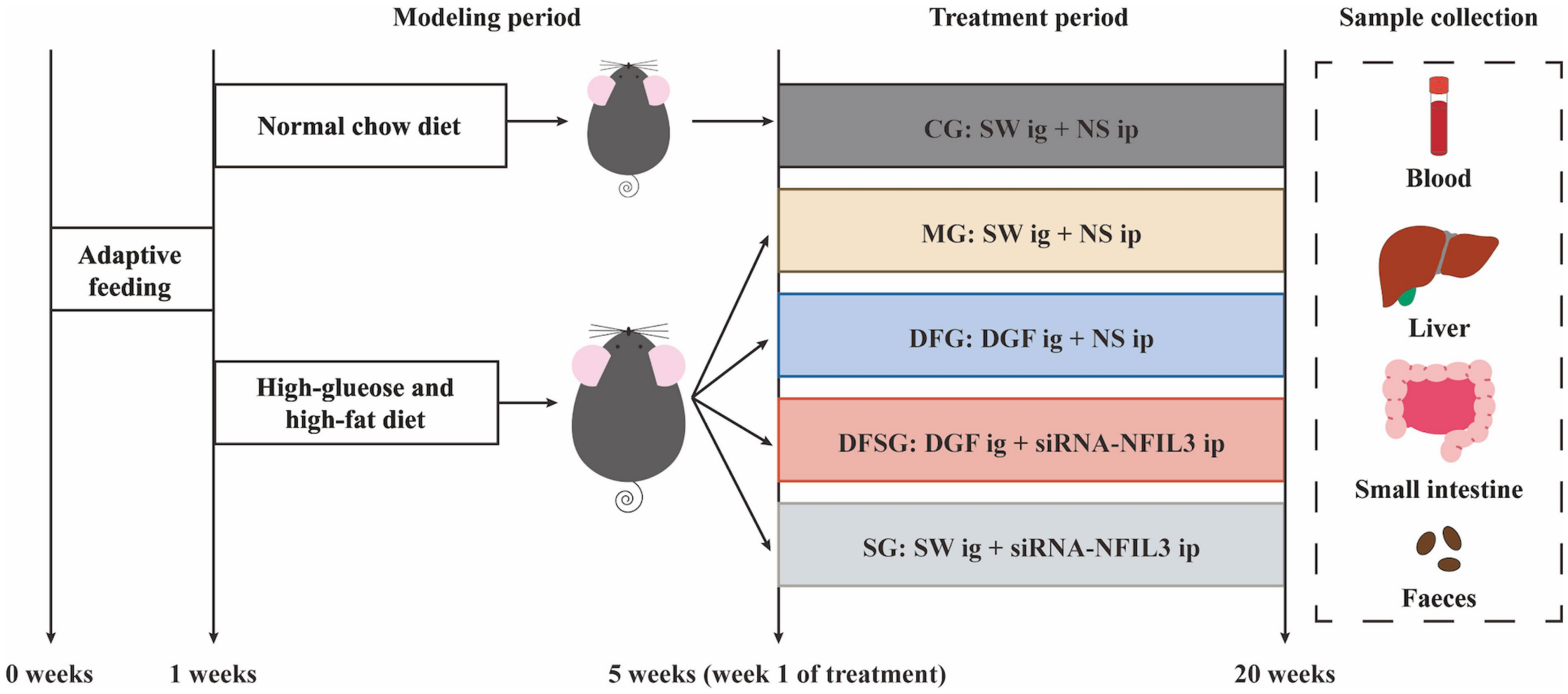
Experimental procedure. CG, Control group; MG, Model group; DFG, Dangua Fang group; DFSG, Dangua Fang + siRNA group; SG, siRNA group; SW, Sterile water; NS, Normal saline; NFIL3, Nuclear factor, interleukin-3 regulated.

Currently, reports on the relationship between GLMD and changes in gut microbiota remain limited, and there is insufficient research on the regulation of gut microbiota and metabolites in GLMD by Traditional Chinese Medicine. Based on this, we established a GLMD mouse model using a high-glucose and high-fat diet to investigate the effects of DGF on gut microbiota and its metabolites. Meanwhile, siRNA-NFIL3 was used as a control to explore the relationship between DGF anti-GLMD effects and those of direct NFIL3 inhibition (Figure 1). This study aims to promote the translation of DGF from basic research to clinical application, thereby enhancing the clinical efficacy of GLMD intervention.

## 2 Materials and methods

### 2.1 Reagents

**Figure 2:**
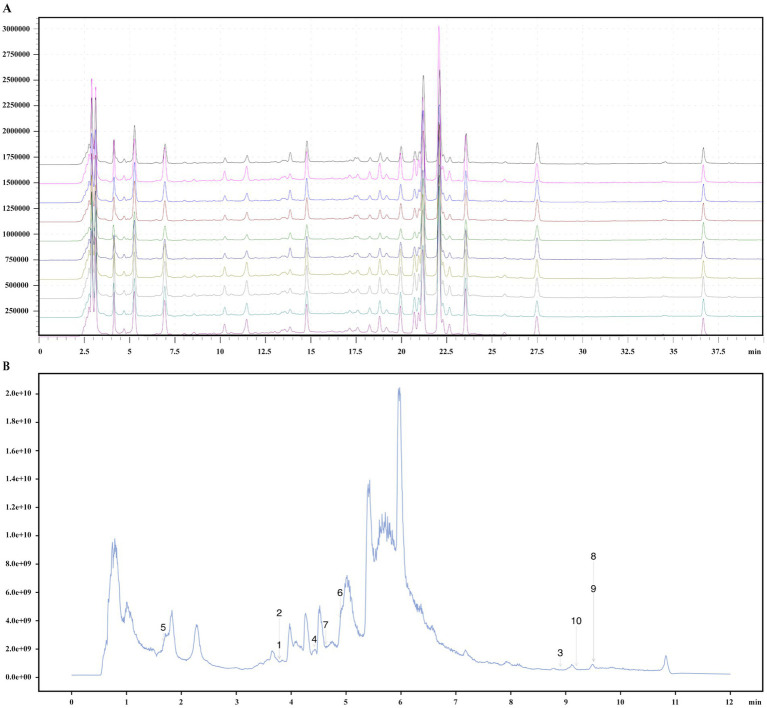
Quality control and chemical composition identification of DGF. **(A)** Fingerprint chromatogram. **(B)** Total ion chromatogram. 1, Pyrocatechol; 2, Hydroquinone; 3, alpha-Linolenic acid; 4, Caffeic acid; 5, Guanosine; 6, Narirutin; 7, Vanillin; 8, Oleic acid; 9, trans-Vaccenic acid; 10, Linoleic acid.

The DGF decoction, commissioned to the Affiliated People’s Hospital of Fujian University of Traditional Chinese Medicine and prepared as a 2 g/mL decoction using a standard production process, was composed of medicinal plants whose names were checked with The World Flora Online on December 20, 2024 (Table 1). The decoction underwent quality control and chemical composition analysis, with preparation and analysis methods detailed in our previous study (Figure 2) (Xianpei et al., 2024; Xianpei et al., 2023). Based on earlier experiments, a daily dose of DGF at 20 g/kg administered via gavage was determined to be the optimal intervention for regulating GLMD in mice (Xianpei et al., 2024; Xianpei et al., 2023; Heng et al., 2022; Heng et al., 2023). 0.9% Sodium chloride injection (Kelun Pharmaceutical Co., Ltd., Nanchang, China, H20083400); siRNA-NFIL3 (Shanghai GenePharma Co., Ltd., Shanghai, China, TS20230428003); ethyl carbamate (Sigma-Aldrich Trading Co., Shanghai, China, PH006652); phosphate-buffered saline (PBS), bicinchoninic acid (BCA) protein assay kit, antibody dilution solution, streptavidin-biotin complex (SABC) immunohistochemistry staining kit, and DAB chromogenic kit (Boster Biological Technology Co., Ltd., Wuhan, China, PYG0072, AR0146, AR1017, SA1020, AR1027); total cholesterol (TC), triglyceride (TG), and free fatty acid (FFA) assay kits (Nanjing Jiancheng Bioengineering Institute, Nanjing, China, A111-2-1, A110-1-1, A042-2-1); mouse NFIL3 enzyme-linked immunosorbent assay (ELISA) kit (Shanghai Westang Bio-Tech, Shanghai, China, FP11773); haematoxylin-eosin (HE) staining solution (Lanjieke Technology Co., Ltd., Beijing, China, BL700B); Masson staining kit and Oil Red O staining solution (Beijing Solarbio Science & Technology Co., Ltd., Beijing, China, G1340, G1261).

**Table 1:** Composition of DGF.

Chinese name	Scientific name	Used parts	Per 100 g (g)
Danshen	*Salvia miltiorrhiza* Bunge	Root	18.75
Gualou	*Trichosanthes kirilowii* Maxim.	Fruit and seeds	18.75
Chuanxiong	Conioselinum anthriscoides ‘Chuanxiong’	Rhizome	12.5
Yujin	*Curcuma longa* L.	Root	12.5
Xiebai	Allium macrostemon Bunge	Bulb	12.5
Chishao	*Paeonia lactiflora* Pall.	Root	12.5
Banxia	*Pinellia ternata* (Thunb.) Makino	Rhizome	6.25
Jiangcan	*Bombyx batryticatus*	Whole insect	6.25

### 2.2 Animals

Thirty-six 7-week-old specific-pathogen-free grade C57 BL/6J mice (weighing 18.76 ± 0.72g) were purchased from Shanghai SLAC Laboratory Animal Co., Ltd. The mice were housed at the Experimental Animal Centre of Fujian University of Traditional Chinese Medicine (approval no.: SYXK (Min) 2023–0004). Housing conditions were as follows: temperature maintained at 22 ± 2°C, daily temperature fluctuation <4°C, relative humidity 50 ± 5%, and a 12 h light/dark cycle. The mice had free access to food and water, and bedding was regularly changed to maintain dryness in the cages. The mice were acclimated to the housing conditions for 1 week. The experimental protocol was approved by the Medical Ethics Committee of the Fujian University of Traditional Chinese Medicine (ethics no.: An 3W2023018).

### 2.3 Model establishment

After 10 h of fasting (water allowed), blood was collected from the tail tip to measure fasting blood glucose (FBG). A 50% glucose solution was then gavaged at 2 g/kg, and the gavage time was recorded. Postprandial blood glucose (PBG) was measured 2 h later. Mice with FBG ≥ 6.1 mmol/L or PBG ≥ 7.8 mmol/L were excluded. Fasting body weights were measured over three consecutive days, and the average weight was used for stratification. Six mice were randomly selected for the control group (CG) and fed a standard diet. The remaining mice were given a high-glucose and high-fat diet containing 70.7% standard feed, 20% sucrose, 7% refined lard, 2% cholesterol, and 0.3% bile salt. After 4 weeks, the fasting body weights were measured again.

### 2.4 Grouping and intervention

The model mice were stratified by body weight and ranked according to their blood glucose levels from high to low and randomly assigned to the model group (MG), DGF group (DFG), DGF + siRNA group (DFSG), or siRNA group (SG) using a random number table, with six mice in each group. Significant differences in body weight were observed between the experimental and CG groups. The DFG and DFSG groups received daily gavages of DGF (20 g/kg), whilst the CG, MG, and SG groups received daily gavages of sterile water of the same volume. Additionally, the DFSG and SG groups received intraperitoneal injections of siRNA-NFIL3 (2.67 mg/kg) every 3 days, whilst the CG, MG, and DFG groups received intraperitoneal injections of normal saline of the same volume every 3 days. These interventions were conducted for 15 weeks (Xianpei et al., 2024; Xianpei et al., 2023; Heng et al., 2022; Heng et al., 2023).

### 2.5 Sample collection

After 10 h of fasting (with water allowed), mouse faeces were collected and stored at −80°C. Mice were anaesthetised using an intraperitoneal injection of 20% ethyl carbamate (5 mL/kg) and placed in a carbon dioxide anaesthesia chamber. Once fully anaesthetised, the abdominal cavity was surgically opened, and blood was collected from the abdominal aorta. The blood samples were left to stand at room temperature for 30 min, then centrifuged at 3,000 rpm for 15 min at 4°C. The supernatant was collected and stored at −80°C. The liver and small intestine tissues were quickly isolated, washed three times with pre-cooled PBS, dried to remove residual moisture, weighed, and photographed. The left lobe of the liver and a 1 cm segment of the lower jejunum were fixed in 4% paraformaldehyde, whilst the remaining liver and intestinal tissues were stored at −80°C.

### 2.6 Experimental methods

#### 2.6.1 Measurement of indicators

The food and water intake, mental state, activity level, and faecal condition of the mice were regularly monitored and recorded throughout the intervention period. Fasting body weight was measured every 2 weeks, whereas FBG and PBG were assessed every 5 weeks. The haemoglobin A1c (HbA1c) levels were measured before sample collection. Liver weight was recorded after sample collection, and the liver index was calculated. Serum TC, TG, and FFA levels were measured using biochemical kits. The NFIL3 levels in the serum, liver, and small intestine were determined using ELISA kits, according to the manufacturer’s instructions.

#### 2.6.2 HE, Masson’s trichrome, and oil red O staining

Liver tissue sections were stained with HE, Masson’s trichrome, and Oil Red O, whereas small intestine sections were stained with HE. For HE staining, sections were de-paraffinised, stained with haematoxylin for 5 min, differentiated with 1% hydrochloric acid alcohol, washed with water to restore blue colour, stained with eosin for 3 min, dehydrated, cleared, and mounted. For Masson’s trichrome staining, sections were de-paraffinised, stained with Weigert’s iron haematoxylin for 7 min, differentiated with acidic ethanol, blued, stained with Ponceau-Fuchsin for 7 min, washed with phosphomolybdic acid and weak acid, stained with aniline blue, dehydrated, cleared, and mounted. For Oil Red O staining, frozen sections (10 μm) were fixed in 10% formalin, immersed in 60% isopropanol, stained with Oil Red O for 15 min, washed, counterstained with Mayer’s haematoxylin for 2 min, and mounted.

#### 2.6.3 Quantitative reverse-transcription PCR (qRT-PCR)

Liver tissues were ground after being frozen in liquid nitrogen, and total RNA was extracted using TRIzol reagent. The RNA concentration and purity were measured using a NanoDrop 2000 spectrophotometer with an A260/A280 ratio of 1.8–2.0. Reverse transcription and PCR were performed according to the manufacturer’s instructions. The primer sequences used were as follows (5′–3′): mus-ACTB-F: AAGATCAAGATCATTGCTCCTCC; mus-ACTB-R: GACTCATCGTACTCCTGCTTGC; mus-NFIL3-F: TCTCAGTGCAGGTGACGAAC; mus-NFIL3-R: AGCCACCGTCTTTGACTTCC, synthesised by Fuzhou Baierman Biotechnology Co., Ltd. The total qPCR volume was 20 μL, and amplification was conducted on an ABI7500 real-time PCR system. ACTB was used for normalisation, and the relative expression levels of the target genes were calculated.

#### 2.6.4 Western blot (WB) analysis

Total protein from mouse liver tissues was extracted using RIPA buffer containing protease and phosphatase inhibitors. The tissues were homogenised at 4°C, centrifuged at 12,000 × g for 10 min, and the supernatant was collected. Protein concentrations were measured using a BCA assay kit. After adding 5 × sodium dodecyl sulfate-polyacrylamide gel electrophoresis (SDS-PAGE) sample buffer, the proteins were heated at 95°C for 5 min. Proteins were separated on a 10% SDS-PAGE gel and transferred to a polyvinylidene fluoride membrane via wet transfer. The membrane was blocked with 5% non-fat milk at room temperature for 1 h and incubated overnight at 4°C with the primary antibody (NFIL3, 1:6,000). The following day, the membrane was washed and incubated with a horseradish peroxidase-conjugated secondary antibody (1:10,000) at room temperature for 1 h. After washing, signals were detected using the Bio-Rad ChemiDoc XRS+ system, and band intensities were analysed using ImageJ software. ACTB was used for normalisation, and the relative expression of the target protein was calculated.

#### 2.6.5 Immunohistochemistry

Paraffin-embedded small intestine tissue sections were de-paraffinised and incubated with 3% hydrogen peroxide at room temperature for 10 min, followed by three washes with PBS. Heat-mediated antigen retrieval was performed using 0.01 M citrate buffer, and sections were blocked with 5% BSA at 37°C for 30 min. The sections were incubated with primary antibodies overnight at 4°C. The following day, after washing with PBS, biotinylated secondary antibodies were added and incubated at 37°C for 30 min. After washing, the sections were incubated with SABC for 30 min, washed with PBS, and developed using DAB. The staining intensity was observed under a microscope and controlled. After washing with water, sections were counterstained with haematoxylin, dehydrated, cleared, and mounted. Image analysis was performed using the ImageJ software.

#### 2.6.6 Metagenomics

Fresh mouse faeces were immediately frozen in liquid nitrogen and transferred to a −80°C freezer for storage. DNA was extracted using the Qiagen QIAamp DNA Stool Mini Kit, following the manufacturer’s instructions. The quality, purity, and concentration of DNA were assessed through agarose gel electrophoresis, a NanoDrop spectrophotometer, and a Qubit fluorometer. Sequencing libraries were constructed using the NEBNext Ultra II DNA Library Prep Kit, which involved DNA fragmentation (ultrasonication to 200–300 bp), end repair, A-tailing, adapter ligation, and PCR amplification. The size and quality of library fragments were assessed using an Agilent 2,100 Bioanalyzer. Qualified libraries were sequenced on an Illumina HiSeq platform with 150 bp paired-end high-throughput sequencing. Raw sequencing data were subjected to quality control, with low-quality reads and adapter sequences removed using FastQC and Trimmomatic software. Quality-controlled data were used for species classification and abundance analysis using Metaphlan2, metagenome assembly using MEGAHIT, and gene function annotation using PROKKA. Functional and metabolic pathway analyses of the gut microbiota were conducted using the Kyoto Encyclopaedia of Genes and Genomes (KEGG).

#### 2.6.7 Untargeted metabolomics

A suitable amount of mouse faeces was mixed with pre-chilled acetonitrile–water, vortexed thoroughly, and incubated on ice for 30 min. The mixture was centrifuged at 12,000 × g for 10 min at 4°C to remove solid residues, and the supernatant was collected and concentrated by vacuum freeze-drying. The dried sample was dissolved in 200 μL of 50% methanol, vortexed, and centrifuged at 12,000 × g for 10 min at 4°C. The supernatant was used for analysis. A pooled sample mixture was prepared as a quality control measure to monitor instrument stability and data reproducibility during the analysis. Metabolites were identified using liquid chromatography–tandem mass spectrometry, with separation optimised using a C18 column and a mobile phase gradient to enhance resolution and sensitivity. Mass spectrometry data were acquired using MassLynx software with a scan range of 50–1,000 m/z. Data processing, including peak detection, alignment, normalisation, and quantification, was performed using Progenesis QI software. Differentially abundant metabolites were identified, and metabolic pathway analyses were performed in conjunction with KEGG.

### 2.7 Statistical analysis

Statistical analysis and visualisation of the experimental data were performed using SPSS 28.0 and GraphPad Prism 10.0. Values conforming to a normal distribution are expressed as mean ± standard deviation. For data with equal variances, a one-way ANOVA followed by the least significant difference (LSD) test was used, whilst for data with unequal variances, a logarithmic transformation was performed before applying the LSD test. Statistical significance was set at *p* < 0.05, *p* < 0.01, and *p* < 0.001.

## 3 Results

### 3.1 Glucolipid metabolism

**Figure 3:**
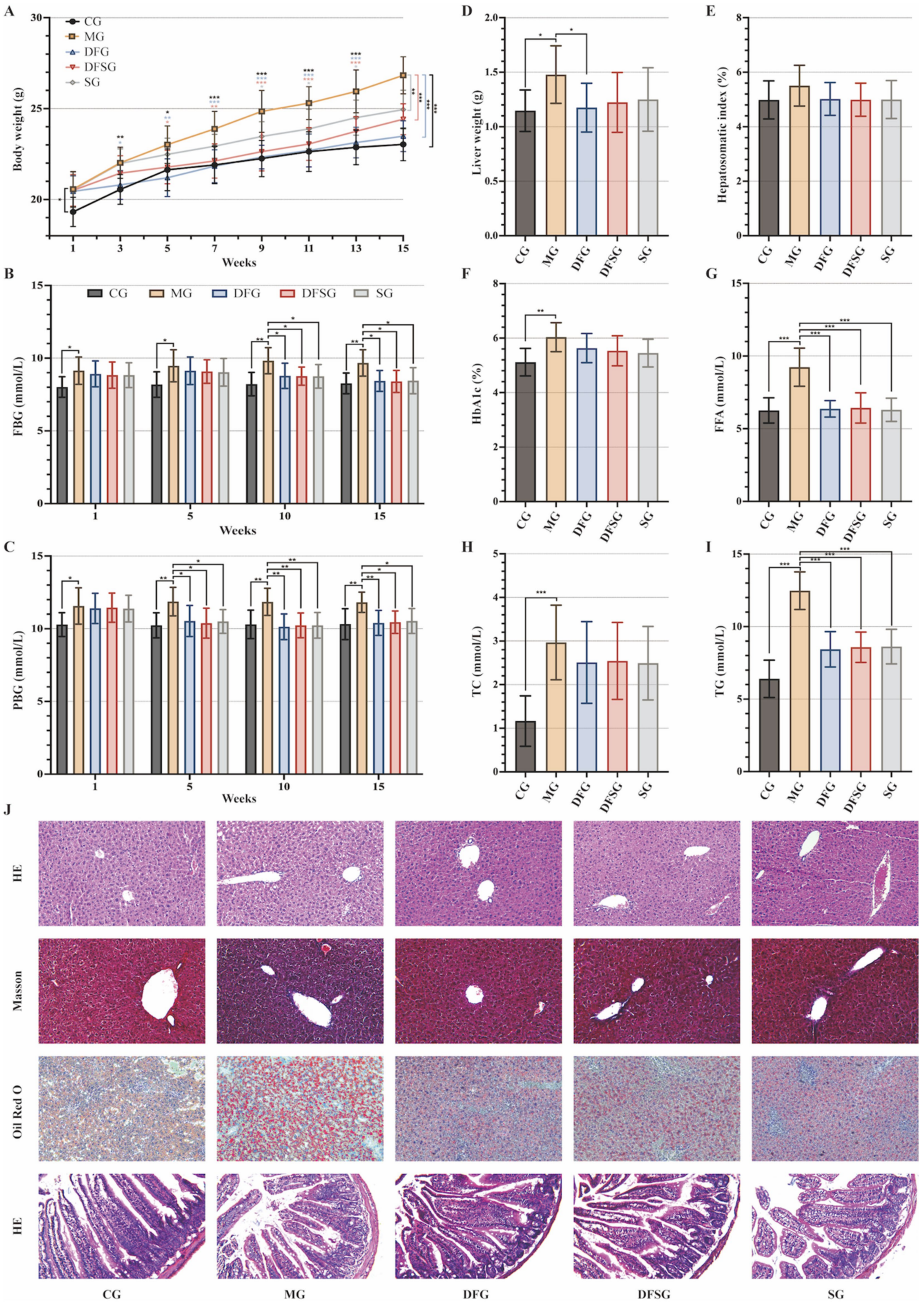
Effects of DGF or NFIL3 inhibition on glucolipid metabolism. **(A)** Body weight changes throughout the intervention period. **(B,C)** FBG and PBG levels during the intervention period. **(D)** Liver weight. **(E)** Hepatosomatic index. **(F)** Blood HbA1c level. **(G–I)** Serum FFA, TC, and TG levels. **(J)** HE, Masson, and Oil Red O staining of liver tissues and small intestine tissues HE staining (magnification: ×200). Data are expressed as mean ± standard deviation. *p < 0.05, **p < 0.01, ***p < 0.001. N = 6 per group. CG, Control group; MG, Model group; DFG, Dangua Fang group; DFSG, Dangua Fang + siRNA group; SG, siRNA group; FBG, Fasting blood glucose; PBG, postprandial blood glucose; HbA1c, Haemoglobin A1c; FFA, Free fatty acid; TC, Total cholesterol; TG, Triglyceride; HE, Haematoxylin-eosin.

The body weight of the MG group was significantly higher than that of the CG group throughout the entire experimental period (*p* < 0.05, *p* < 0.01, and *p* < 0.001). The body weights of the DFG group from weeks 3 to 15, the DFSG group from weeks 5 to 15, and the SG group from weeks 9 to 15 were significantly lower than those of the MG group (*p* < 0.05, *p* < 0.01, and *p* < 0.001). Compared with the DFG group, the body weight of the SG group was significantly higher from weeks 3 to 15 (*p* < 0.05) (Figure 3A). The FBG and PBG levels in the MG group were consistently higher than those in the CG group (*p* < 0.05 and *p* < 0.01). The FBG levels in the DFG, DFSG, and SG groups at weeks 10 and 15, as well as the PBG levels at weeks 5, 10, and 12, were significantly lower than those in the MG group (*p* < 0.05 and *p* < 0.01). No significant differences in blood glucose changes were observed between the intervention groups (Figures 3B,C). The liver weight in the MG group was significantly higher than that in the CG group (*p* < 0.05), whilst the DFG group had significantly lower liver weight than the MG group (*p* < 0.05) (Figure 3D). No statistical differences in the liver index were observed among the groups (Figure 3E). The levels of HbA1c, FFA, TC, and TG in the MG group were significantly higher than those in the CG group (*p* < 0.01 and *p* < 0.001). The FFA and TG levels in the DFG, DFSG, and SG groups were significantly lower than those in the MG group (*p* < 0.001), whereas no significant differences in HbA1c and TC were observed. Additionally, no statistically significant differences in these indicators were observed among the three intervention groups (Figures 3F–I).

HE staining of liver tissues showed that the hepatocytes in the CG group were arranged orderly with centrally located nuclei, clear lobular structures, and radiating hepatic cords. In the MG group, hepatocyte volume increased, the cells were densely packed with more rod-shaped nuclei, and the hepatic cords were disorganised, lacking a radiating structure, accompanied by notable inflammatory cell infiltration. In the DFG, DFSG, and SG groups, the hepatocytes exhibited regular morphology, increased intercellular space, a clear lobular structure, radiating hepatic cords, and minimal inflammatory cell infiltration. Masson staining indicated a small amount of blue collagen fibres in the portal area of the CG group, whereas the MG, DFG, DFSG, and SG groups showed greater blue collagen fibre deposition in the portal area, Disse’s space, and around some hepatocytes, with the most extensive collagen deposition observed in the MG group. Oil Red O staining revealed scattered granular red lipid droplets in the CG group, whereas varying amounts and sizes of red lipid droplets were observed in the MG, DFG, DFSG, and SG groups, with the MG group exhibiting the highest accumulation of lipid droplets. HE staining of the small intestinal mucosa showed a clear and regular structure with neatly arranged villi in the CG group, without congestion, oedema, or inflammatory infiltration. In contrast, the MG group exhibited a blurred small intestinal mucosal structure, fused adjacent villi, villus damage, fracture, shedding, numerous goblet cells, and inflammatory infiltration. In the DFG, DFSG, and SG groups, the mucosal structure was clear, with neatly arranged villi, minimal villi damage, fracture, and shedding, and a small number of goblet cells, with no obvious congestion, oedema, or inflammatory infiltration observed (Figure 3J).

In summary, we established a GLMD mouse model and confirmed that DGF and NFIL3 inhibition could significantly regulate glucolipid metabolism in the model mice, ameliorate pathological damage to the liver and small intestine, and effectively alleviate GLMD. However, DGF exhibited the most pronounced reduction in body and liver weights in the model mice, whereas the combined treatment did not show a superior effect compared with DGF alone.

### 3.2 NFIL3 expression in the serum, liver, and small intestine

**Figure 4:**
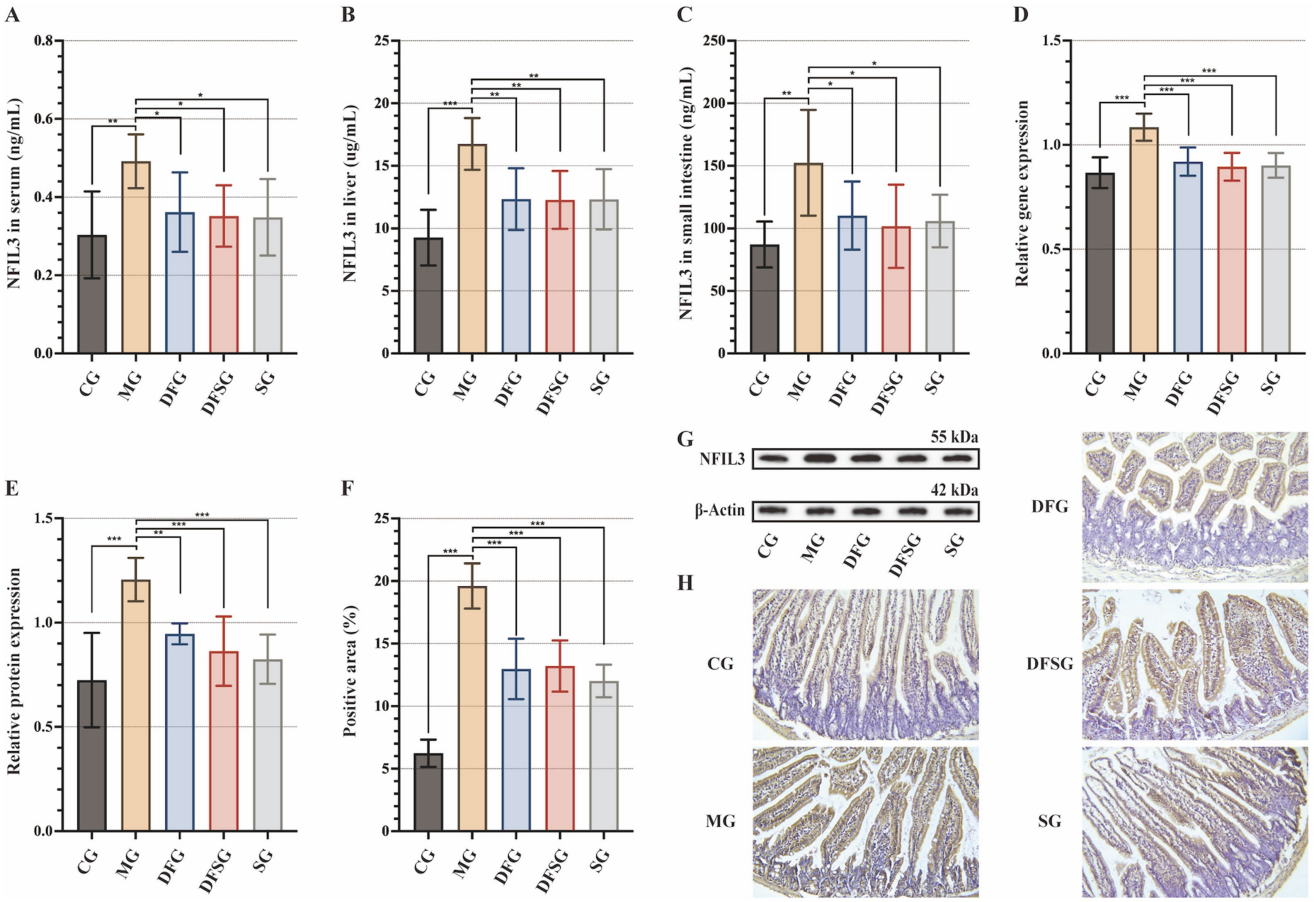
Effects of DGF or NFIL3 inhibition on NFIL3. **(A–C)** NFIL3 levels in the serum, liver, and small intestine. **(D)** NFIL3 mRNA levels in the liver. **(E,G)** NFIL3 protein levels in the liver. **(F)** Positive area of NFIL3. **(H)** Immunohistochemistry of small intestine (magnification: ×200). Data are expressed as mean ± standard deviation. ^*^p < 0.05, ^**^p < 0.01, ^***^p < 0.001. *N* = 6 per group. NFIL3, Nuclear factor, interleukin-3 regulated; CG, Control group; MG, Model group; DFG, Dangua Fang group; DFSG, Dangua Fang + siRNA group; SG, siRNA group.

Compared with the CG group, NFIL3 levels in the serum, liver, and small intestine of the MG group were significantly elevated (*p* < 0.01 and *p* < 0.001). Moreover, NFIL3 levels in the serum, liver, and small intestine were significantly reduced in the DFG, DFSG, and SG groups, compared with those in the MG group (*p* < 0.05 and *p* < 0.01), but no significant differences were observed among these three groups (Figures 4A–C). The mRNA and protein expression levels of NFIL3 in the liver tissues of the MG group were significantly higher than those in the CG group (*p* < 0.001). However, the mRNA and protein expression levels of NFIL3 in the DFG, DFSG, and SG groups were significantly lower than those in the MG group (*p* < 0.01 and *p* < 0.001), with no significant differences observed among the three groups (Figures 4D,E,G). Immunohistochemical analysis showed that NFIL3 expression in the small intestine of the MG group was significantly higher than that in the CG group (*p* < 0.001). In contrast, NFIL3 expression in the DFG, DFSG, and SG groups was significantly lower than that in the MG group (*p* < 0.001), but there were no significant differences observed among the three groups (Figures 4F,H).

These results suggest that DGF reduces NFIL3 expression in the serum, liver, and small intestine of model mice, with effects similar to those of siRNA-NFIL3. However, no evident synergistic effect was observed when both treatments were administered together.

### 3.3 Differential microbiota and linear regression analysis of metabolic indicators

**Figure 5:**
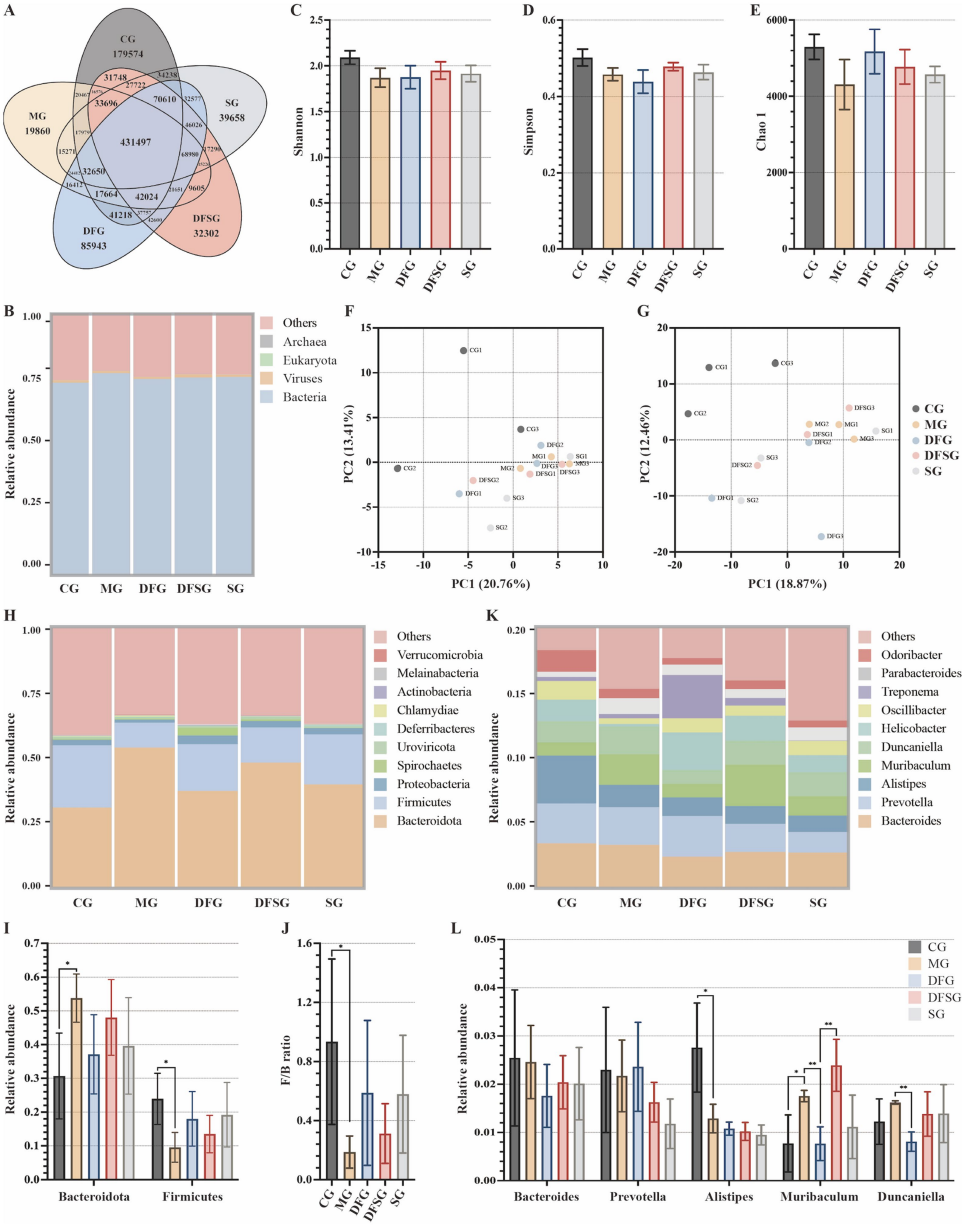
Effects of DGF or NFIL3 inhibition on the gut microbiota. **(A)** Number of genes. **(B)** Relative abundance of species at the kingdom level. **(C–E)** Shannon, Simpson, and Chao 1 indices based on species abundance. **(F,G)** PCA results based on species abundance at the phylum and genus levels. **(H)** Relative abundance of species at the phylum level. **(I)** Relative abundance of Bacteroidota and Firmicutes. **(J)** Ratio of Firmicutes to Bacteroidota. **(K)** Relative abundance of species at the genus level. **(L)** Relative abundance of Bacteroides, Prevotella, Alistipes, Muribaculum and Duncaniella. Data are expressed as mean ± standard deviation. ^*^p < 0.05. *N* = 3 per group. CG, Control group; MG, Model group; DFG, Dangua Fang group; DFSG, Dangua Fang + siRNA group; SG, siRNA group; PCA, Principal component analysis.

The petal diagram of gene numbers (Figure 5A) revealed that 431,497 common genes were present in the CG, MG, DFG, DFSG, and SG groups. The CG group had 179,574 unique genes, the MG group had 19,860, the DFG group had 85,943, the DFSG group had 32,302, and the SG group had 39,658. The gut microbiota of each group included bacteria, viruses, eukaryotes, and archaea, with bacteria comprising 75% of the total, detailed species information is available in the Supplementary materials (Figure 5B). Alpha diversity index analysis (Figures 5C–E) showed no significant differences in the Shannon, Simpson, and Chao1 indices among the groups. Principal component analysis based on species richness (Figures 5F,G) indicated a clear separation between the groups at the phylum and genus levels, suggesting differences in the gut microbiota composition.

The relative abundance histogram of the species (Figure 5H) illustrated that, at the phylum level, Bacteroidota and Firmicutes dominated across all groups, with a relative abundance exceeding 50%, followed by Proteobacteria and Spirochaetes. Compared with the CG group, the relative abundance of Bacteroidota in the MG group was significantly higher (*p* < 0.05), whereas the relative abundances of Firmicutes (*p* < 0.05) and Proteobacteria were lower. Compared with the MG group, the relative abundance of Bacteroidota decreased, whereas the abundances of Firmicutes and Proteobacteria increased in the DFG, DFSG, and SG groups, although these changes were not significant (Figure 5I). Furthermore, the Firmicutes/Bacteroidota ratio in the MG group was lower than that in the CG group (*p* < 0.05), whilst the ratio in the DFG, DFSG, and SG groups was higher than that in the MG group, but the differences were not statistically significant (Figure 5J). At the genus level, compared with the CG group, the relative abundances of Bacteroides, Prevotella, and Alistipes (*p* < 0.05) were lower in the MG group, whilst those of Muribaculum and Duncaniella increased. After intervention, the relative abundances of these species changed to varying degrees across the groups (Figures 5K,L).

**Figure 6:**
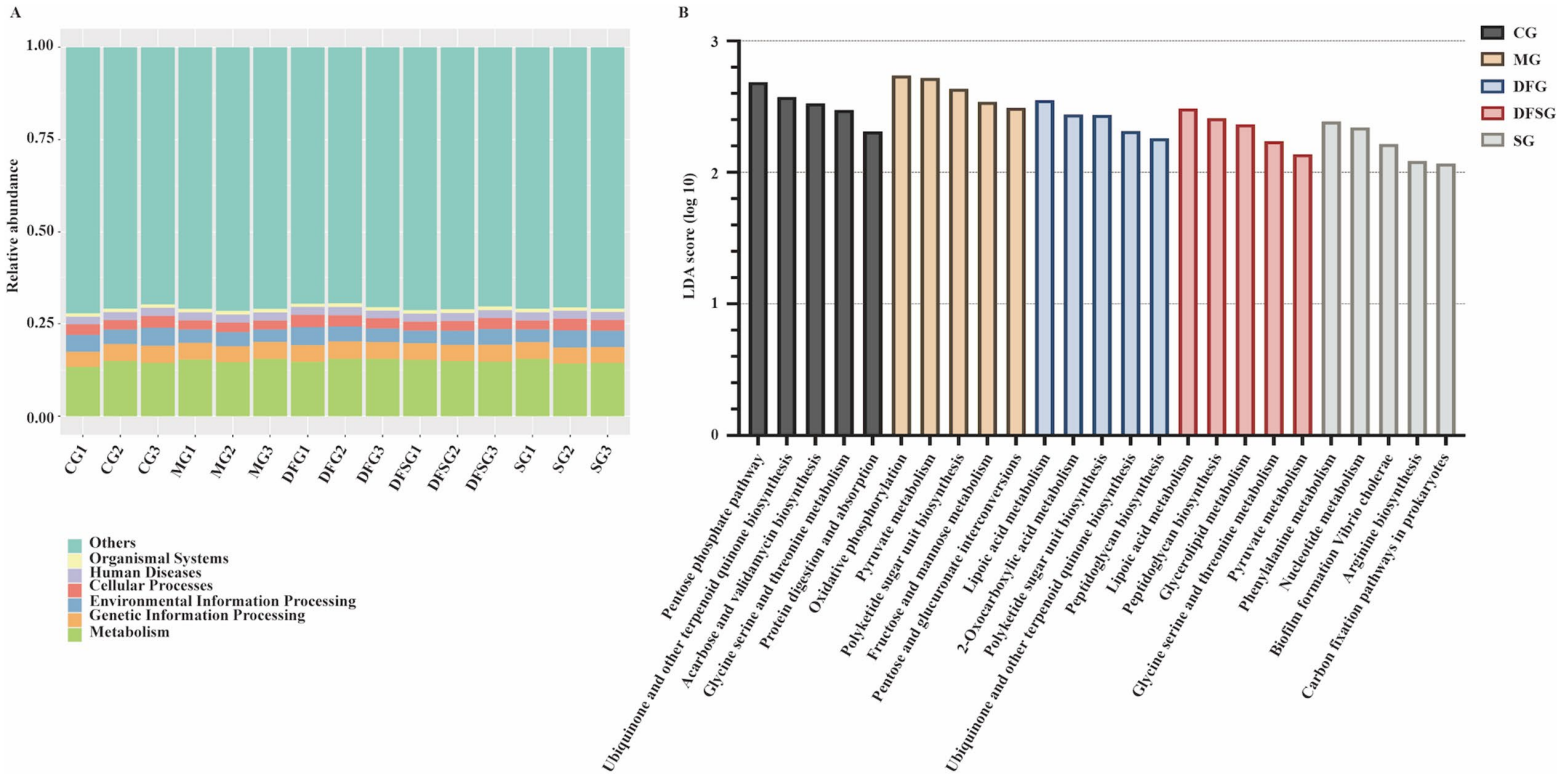
Potential functions of differential microbiota. **(A)** Relative abundance of functional annotations at level 1. **(B)** LDA scores for differential functions. *N* = 3 per group. CG, Control group; MG, Model group; DFG, Dangua Fang group; DFSG, Dangua Fang + siRNA group; SG, siRNA group; LDA, Linear Discriminant Analysis.

Considering that changes in gut microbiota are closely linked to alterations in their functions, we analysed the potential functions associated with the differential microbiota. Samples in this study identified 6, 48, and 441 pathways at KEGG levels 1, 2, and 3, respectively. Functional genes of the gut microbiota in each group were annotated to six metabolic pathways, with the metabolic pathway showing the highest relative abundance, accounting for over 12.5% (Figure 6A). The distribution histogram of LDA scores for differential functions (Figure 6B) showed that the significantly different potential functions between the MG and CG groups included oxidative phosphorylation, pyruvate metabolism, polyketide sugar unit biosynthesis, fructose and mannose metabolism, and pentose and glucuronate interconversions. The significantly different potential functions between the DFG and MG groups included lipoic acid metabolism, 2-oxocarboxylic acid metabolism, polyketide sugar unit biosynthesis, ubiquinone and other terpenoid quinone biosynthesis, and peptidoglycan biosynthesis. The significantly different potential functions between the DFSG and MG groups included lipoic acid metabolism, peptidoglycan biosynthesis, glycerolipid metabolism, glycine serine and threonine metabolism, and pyruvate metabolism. The significantly different potential functions between the SG and CG groups included pyrimidine metabolism, nucleotide metabolism, biofilm formation *vibrio cholerae*, arginine biosynthesis, and carbon fixation pathways in prokaryotes.

Linear regression analysis was conducted using the relative abundances of Bacteroidota and Firmicutes as independent variables, with body weight, liver weight, and the levels of FBG, PBG, HbA1c, TC, TG, FFA, and NFIL3 as dependent variables. The results showed that Bacteroidota significantly influenced PBG levels in mice (*p* < 0.05). Additionally, Bacteroidota and Firmicutes significantly affected TC, TG, and FFA levels (*p* < 0.05). Although some associations were observed for other indicators, they did not reach statistical significance (Table 2).

**Table 2:** Linear regression analysis results.

Variable	Bacteroidota				Firmicutes			
	*F*	*b*	*t*	*p*	*F*	*b*	*t*	*p*
Body weight	3.00	0.43	1.73	0.11	2.96	−0.43	−1.72	0.11
Liver weight	0.42	0.18	0.65	0.53	0.48	−0.19	−0.70	0.50
FBG	2.62	0.41	1.62	0.13	1.42	−0.31	−1.19	0.26
OGTT 2 h BG	4.84	0.52	2.20	0.04^*^	1.95	−0.36	−1.40	0.19
HbA1c	0.07	0.07	0.26	0.80	0.03	−0.05	−0.17	0.87
TC	9.34	0.65	3.06	0.01^*^	8.53	−0.63	−2.92	0.01^*^
TG	7.43	0.60	2.73	0.02^*^	7.24	−0.60	−2.69	0.02^*^
FFA	5.65	0.55	2.38	0.03^*^	5.48	−0.55	−2.34	0.04^*^
NFIL3 in serum	1.94	0.36	1.39	0.19	1.28	−0.30	−1.13	0.28
NFIL3 in liver	2.56	0.41	1.56	0.13	3.51	−0.46	−1.87	0.08
NFIL3 in small intestine	2.93	0.43	1.71	0.11	3.42	−0.46	−1.85	0.09

Data are expressed as mean ± standard deviation. ^*^p < 0.05. *N* = 3 per group. FBG, Fasting blood glucose; OGTT 2 h BG, OGTT 2-h blood glucose; HbA1c, Haemoglobin A1c; TC, Total cholesterol; TG, Triglyceride; FFA, Free fatty acid; NFIL3, Nuclear factor, interleukin-3 regulated.

### 3.4 Changes in differential metabolites and their associated metabolic pathways

**Figure 7:**
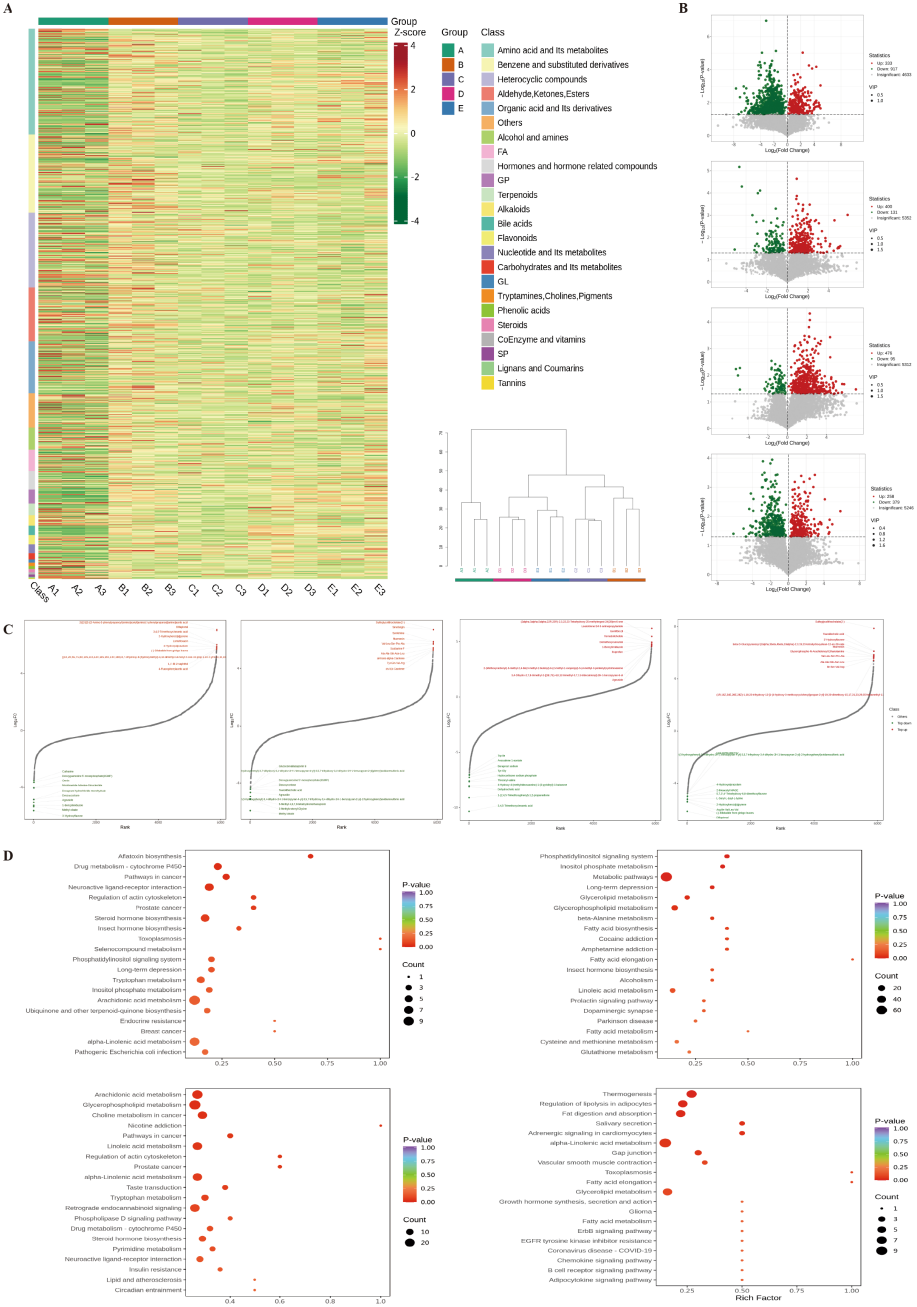
Effects of DGF or NFIL3 inhibition on metabolites. **(A)** Clustering heatmap and hierarchical clustering tree of differential metabolites. **(B)** Volcano plots of differential metabolites. **(C)** Dynamic distribution plots of differential metabolite content. **(D)** KEGG enrichment plots of differential metabolites. *N* = 3 per group. KEGG, Kyoto Encyclopaedia of Genes and Genomes.

The clustering heatmap and hierarchical clustering tree of differential metabolites (Figure 7A) revealed significant differences in metabolites among the groups, mainly encompassing amino acids and their metabolites, benzene and its substituted derivatives, heterocyclic compounds, aldehydes, ketones, esters, and organic acids and their derivatives. Volcano plots (Figure 7B) show that, compared with the CG group, 333 metabolites were significantly upregulated, and 917 metabolites were significantly downregulated in the MG group. Compared with the MG group, 400 metabolites were significantly upregulated, and 131 were downregulated in the DFG group, whereas 476 metabolites were significantly upregulated, and 95 were downregulated in the SG group. Compared with the DFG group, 258 metabolites were significantly upregulated, and 379 metabolites were significantly downregulated in the SG group.

The dynamic distribution of differential metabolite levels (Figure 7C) revealed that, compared with the CG group, metabolites such as leukotriene B4-3-aminopropylamide, (2alpha,3alpha,5alpha,22R,23R)-2,3,22,23-tetrahydroxy-25-methylergost-24(28)en-6-one, and gemfibrozil were significantly upregulated in the MG group. Conversely, metabolites such as 3,4,5-trimethoxycinnamic acid, 1-(2,4,5-trimethoxyphenyl)-1,2-propanedione, dehydrocholic acid, and 4-hydroxy-4-(methylnitrosamino)-1-(3-pyridinyl)-1-butanone were significantly downregulated. Compared with the MG group, metabolites such as sulfoglycolithocholate(2-), sinefungin, and senkirkine were significantly upregulated in the DFG group, whilst methyl oleate, 3-methylcrotonyl glycine, and 5-methyl-5,6,7,8-tetrahydromethanopterin were significantly downregulated. Compared with the MG group, metabolites such as 2-[[2-[[2-[(2-amino-3-phenylpropanoyl)amino]acetyl]amino]-3-phenylpropanoyl]amino]acetic acid, dillapional, and 3,4,5-trimethoxycinnamic acid were significantly upregulated in the SG group, whilst 3′-hydroxyflavone, methyl oleate, and 1-benzylimidazole were significantly downregulated. Compared with the DFG group, metabolites such as sulfoglycolithocholate(2-), taurallocholic acid, and 3′-hydroxyflavone were significantly upregulated in the SG group. Conversely, metabolites such as dillapiona, (−)-bilobalide from ginkgo leaves, and arg-ile-val-leu-va were significantly downregulated.

Annotation of differential metabolites using the KEGG database (Figure 7D) indicated that compared with the CG group, the MG group exhibited significant differences in metabolic pathways, including arachidonic acid metabolism, glycerophospholipid metabolism, linoleic acid metabolism, alpha-linolenic acid metabolism, and lipid and atherosclerosis. Compared with the MG group, the DFG group showed significant differences in metabolic pathways involving glycerolipid metabolism, glycerophospholipid metabolism, fatty acid biosynthesis, fatty acid elongation, and fatty acid metabolism. Compared with the MG group, the SG group showed significant differences in pathways such as steroid hormone biosynthesis, phosphatidylinositol signalling system, tryptophan metabolism, inositol phosphate metabolism, and alpha-linolenic acid metabolism. Compared with the DFG group, the SG group showed significant differences in metabolic pathways involving thermogenesis, regulation of lipolysis in adipocytes, fat digestion and absorption, alpha−linolenic acid metabolism, and glycerolipid metabolism.

### 3.5 Correlation analysis between gut microbial species and differential metabolites

**Figure 8:**
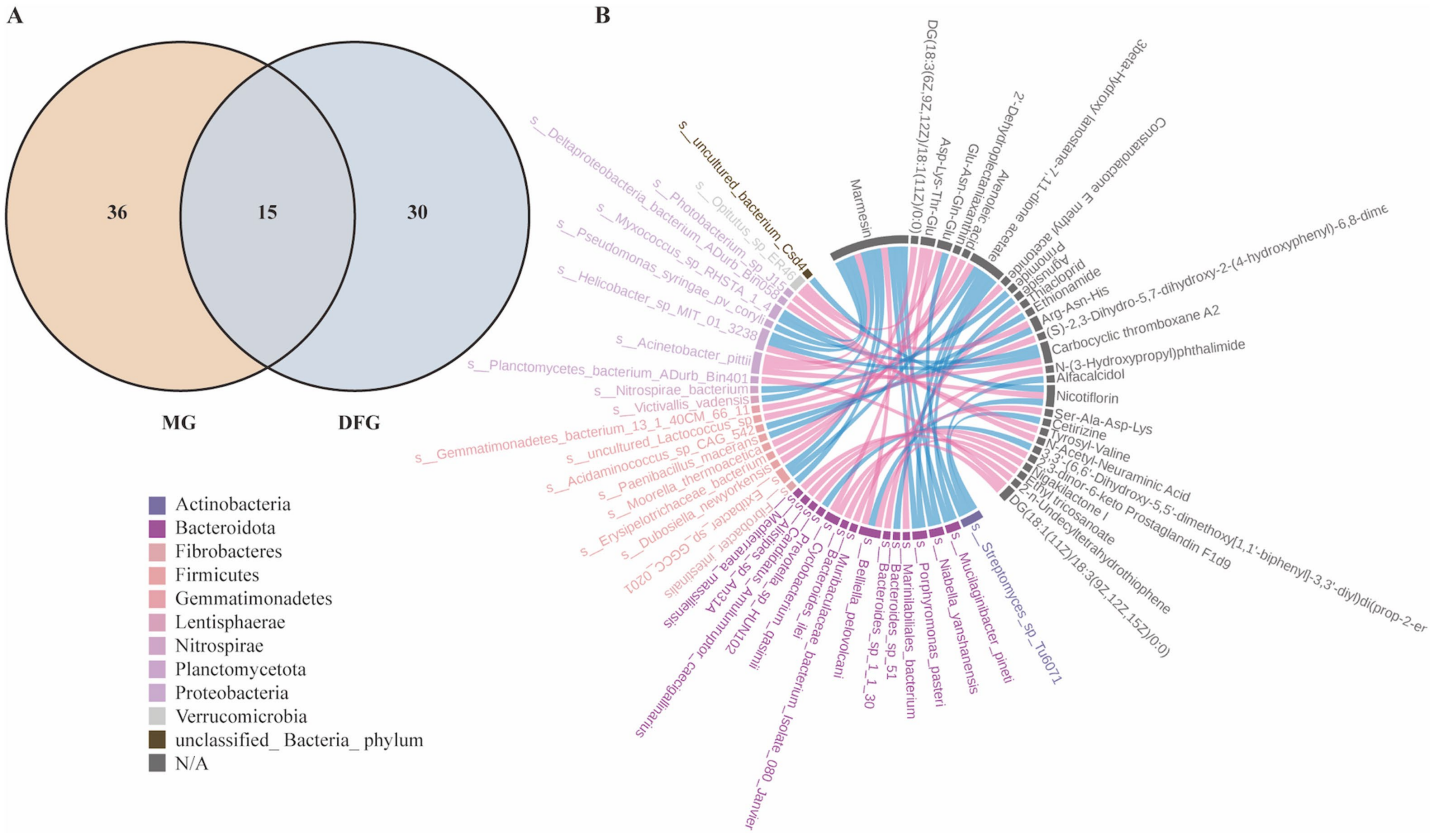
Correlation between gut microbial species and differential metabolites. **(A)** Common KEGG pathways. **(B)** Chord diagram of the correlation between differential microorganisms (species level) and differential metabolites. *N* = 3 per group. MG, Model group; DFG, Dangua Fang group; KEGG, Kyoto Encyclopaedia of Genes and Genomes.

A combined analysis of metagenomics and untargeted metabolomics was conducted to explore the correlation between gut microbial species and differential metabolites in mice. The Venn diagram (Figure 8A) illustrates that 15 pathways exhibited differences in metagenomics and untargeted metabolomics between the MG and DFG groups. Among these, the metabolism-related pathways included pentose and glucuronate interconversion, fatty acid elongation, lipoic acid metabolism, phenylalanine metabolism, and glycerolipid metabolism. Subsequently, a chord diagram was constructed to depict significant relationships between the gut microbiota (at the species level) and metabolites based on Pearson correlation coefficients of less than −0.8 or greater than 0.8, with *p* < 0.05. As shown (Figure 8B), each metabolite was significantly correlated with multiple bacterial species.

The above results indicate that GLMD was associated with alterations in the gut microbiota and their metabolites. DGF and NFIL3 inhibition modulate the gut microbiota and their metabolites in model mice, reversing the dysbiosis induced by a high-glucose and high-fat diet through altered lipid metabolism-related pathways, with similar effects.

## 4 Discussion

Excessive intestinal fat absorption is a key pathogenic factor in GLMD (Zhang et al., 2023). As a vital digestive organ, the intestine not only plays a significant role in nutrient absorption and metabolism but also provides an ecological environment for the survival and reproduction of numerous microorganisms (Schneider et al., 2024; Shalon et al., 2023). The intestinal environment is influenced by various physical and chemical factors, including diet, medication, lifestyle, and disease states (Ma et al., 2024). The composition and function of the gut microbiota are influenced by changes in the intestinal environment (Baars et al., 2024; Ullah et al., 2024; Wang et al., 2024). For example, an increased pH in the small intestine promotes the growth of beneficial bacteria, such as Bifidobacterium and Lactobacillus, whereas a decreased pH in the colon favours the growth of anaerobic bacteria, such as Bacteroides and Prevotella (Rivière et al., 2016; Tett et al., 2019; Koh et al., 2016). A Western diet, high in animal proteins, sugars, and fats, significantly increases the abundance of the Bacteroides and Ruminococcus phyla (Yatsunenko et al., 2012; Mukhopadhya et al., 2012), contributing to a higher prevalence of metabolic diseases such as obesity, fatty liver disease, diabetes, and atherosclerosis among Western populations (Perler et al., 2023).

Regulating the gut environment and microbiota has been demonstrated to be beneficial for treating metabolic diseases (Baars et al., 2024). Metformin is known to exert its antidiabetic effects by increasing the abundance of bacteria associated with short-chain fatty acid production and mucin degradation (Gao et al., 2024). In mice, transplanting the gut microbiota from patients treated with metformin into germ-free mice improved their glucose intolerance (Forslund et al., 2015). Acarbose reduces lipopolysaccharide and inflammatory factor levels in patients with type 2 diabetes by altering microbial fermentation processes through the inhibition of carbohydrate hydrolysis (Zhang et al., 2019). Additionally, traditional Chinese medicines, such as Shugan decoction, improve the intestinal ecological environment in rats by regulating faecal pellet output and visceral sensitivity, thereby increasing the abundance of beneficial bacteria (Hang et al., 2022). Angelica polysaccharides can alter the structure and function of the gut microbiota by modulating L-cysteine levels, thereby alleviating HFD-induced abnormal blood glucose levels in mice (Tang et al., 2023). Our study demonstrated that the structure and function of the gut microbiota in GLMD model mice induced by a high-glucose and high-fat diet were altered, accompanied by elevated NFIL3 expression.

NFIL3 is a basic leucine zipper transcription factor involved in immune regulation, circadian rhythms, and *de novo* lipogenesis (Wang et al., 2017). NFIL3 overexpression in the liver promotes gluconeogenesis, disrupts lipid metabolism and insulin signalling, induces insulin resistance in the liver and muscles, and increases the risk of metabolic syndrome (Matsumura et al., 2021). Targeted knockout of the NFIL3 gene significantly enhances the activity of adenosine monophosphate-activated protein kinase (AMPK) in the liver and increases the abundance of the AMPK β1 subunit, thereby improving hepatic steatosis and liver function in mice (Wang et al., 2023; Yang et al., 2020; Wu et al., 2024). Furthermore, the gut microbiota is known to influence NFIL3 expression through metabolites, immune cell regulation, and circadian rhythms (Wang et al., 2017; Kubo, 2020; Lin et al., 2024). Intestinal epithelial cells sense symbiotic bacteria through Toll-like receptors and the signalling adaptor myeloid differentiation primary response 88, which in turn, regulates NFIL3 expression. NFIL3 subsequently modulates dietary fatty acid transport proteins, fatty acid-binding proteins, and fatty acid hydroxylases, thereby playing a role in host metabolism (Kubo, 2020). Meanwhile, the gut microbiota can interact with immune cells in the small intestine through secretions, activating signalling pathways that inhibit nuclear receptor subfamily 1 group D member 1 protein in intestinal cells, indirectly increasing NFIL3 expression (Wang et al., 2017).

Our study demonstrated that both DGF and NFIL3 inhibition effectively alleviated GLMD in model mice by significantly reducing NFIL3 expression in the serum, liver, and small intestine. Additionally, these interventions decreased the relative abundance of Bacteroidota and increased the abundances of Firmicutes and Proteobacteria at the phylum level. Bacteroidota and Firmicutes are the primary components of the gut microbiota, accounting for up to 90% of its population (Human Microbiome Project Consortium, 2012). Bacteroidota are closely linked to human metabolic diseases and provide energy to the host by fermenting indigestible polysaccharides (Johnson et al., 2017). Compared with Firmicutes, Bacteroidota encode more carbohydrate-active enzymes and signal peptides that facilitate the degradation of glycans, which are difficult to penetrate the bacterial cell wall (Bäckhed et al., 2005; El Kaoutari et al., 2013). Diabetes is associated with an increased abundance of Bacteroidota, which in turn, can reduce insulin sensitivity and promote fat accumulation in mice by affecting short-chain fatty acid metabolism pathways (Qin et al., 2012; Boulangé et al., 2016). Additionally, Proteobacteria are beneficial in lowering blood glucose levels and improving insulin resistance (Tsai et al., 2019). Supplementing type 2 diabetic mice with probiotics increases the abundance of Proteobacteria and reduces FBG levels (Salles et al., 2020).

The ratio of Firmicutes to Bacteroidota was once considered a marker of obesity (De Bandt et al., 2011; Zou et al., 2020). However, this relationship has not been consistently observed. Our study found that the Firmicutes to Bacteroidota ratio in the MG group was lower than that in the CG group, but it increased following the intervention. A survey of tribal populations in southern India reported that, owing to dietary restrictions related to religion and culture, both the tribal population and individuals following a normal diet in the same region exhibited high Firmicutes and low Bacteroidota abundances despite being lean (Ramadass et al., 2017). Meta-analyses also suggest that there is no significant difference in the Firmicutes to Bacteroidota ratio between normal-weight and obese individuals (Sze and Schloss, 2016; Walters et al., 2014). These contradictory results may be linked to factors such as varying lifestyles, antibiotic abuse, food additive intake, and exposure to pollutants. Therefore, caution should be exercised when using the Firmicutes to Bacteroidota ratio as a health indicator (Magne et al., 2020).

Based on our findings, we propose that DGF alters the intestinal lipid environment in mice by regulating NFIL3 and the gut microbiota, with anti-GLMD effects similar to those of direct NFIL3 inhibition. To verify this, we conducted a linear regression analysis of the differential gut microbial species and metabolism-related indicators, which further confirmed that changes in blood glucose and lipid levels in mice were significantly influenced by Bacteroidota and Firmicutes. Additionally, KEGG database annotation of metabolites in the faeces of mice following DGF or siRNA-NFIL3 intervention revealed multiple pathways enriched in lipid metabolism, including glycerolipid metabolism, fatty acid metabolism, fatty acid biosynthesis, fatty acid elongation, steroid hormone biosynthesis, the phosphatidylinositol signalling system, arachidonic acid metabolism, and alpha-linolenic acid metabolism. This further supports the notion that changes in the gut microbiota induced by DGF or NFIL3 inhibition, lead to alterations in lipid metabolism-related pathways, reinforcing the link between DGF regulation of NFIL3 and the gut microbiota, and changes in lipid metabolism.

## 5 Conclusion

Our study validates that changes in the gut microbiota and the intestinal lipid environment are closely associated with GLMD. Direct NFIL3 inhibition can regulate gut microbiota dysbiosis in GLMD mice. DGF reduces NFIL3 expression in model mice, modulates the gut microbiota and their metabolites, and alters lipid metabolism-related pathways. Its anti-GLMD effects may be achieved through the inhibition of NFIL3. In future studies, we aim to further elucidate the mechanisms underlying DGF intervention in GLMD and optimise its potential for clinical application.

## Data Availability

The datasets presented in this study can be found in online repositories. The names of the repository/repositories and accession number(s) can be found in the article/Supplementary material.

## Glossary

DGF: Dangua Fang
NFIL3: Nuclear factor, interleukin-3 regulated
GLMD: Glucolipid metabolism disorder
CG: Control group
MG: Model group
DFG: Dangua Fang group
DFSG: Dangua Fang + siRNA group
SG: siRNA group;
HFD: High-fat diet;
PBS: Phosphate-buffered saline
TC: Total cholesterol
TG: Triglyceride
FFA: Free fatty acid
ELISA: Enzyme-linked immunosorbent assay
HE: Haematoxylin-eosin
BCA: Bicinchoninic acid
SABC: Streptavidin-biotin complex
FBG: Fasting blood glucose
PBG: postprandial blood glucose
HbA1c: Haemoglobin A1c
qRT-PCR: Quantitative reverse-transcription PCR
WB: Western blot
SDS-PAGE: Sulfate-polyacrylamide gel electrophoresis
KEGG: Kyoto Encyclopaedia of Genes and Genomes
PCA: Principal component analysis
AMPK: AMP-activated protein kinase

## References

[ref1] Baars D. P. Fondevila M. F. Meijnikman A. S. Nieuwdorp M. (2024). The central role of the gut microbiota in the pathophysiology and management of type 2 diabetes. Cell Host Microbe 32, 1280–1300. doi: 10.1016/j.chom.2024.07.017, PMID: 39146799

[ref2] Bäckhed F. Ley R. E. Sonnenburg J. L. Peterson D. A. Gordon J. I. (2005). Host-bacterial mutualism in the human intestine. Science 307, 1915–1920. doi: 10.1126/science.1104816, PMID: 15790844

[ref3] Boulangé C. L. Neves A. L. Chilloux J. Nicholson J. K. Dumas M. E. (2016). Impact of the gut microbiota on inflammation, obesity, and metabolic disease. Genome Med. 8:42. doi: 10.1186/s13073-016-0303-2, PMID: 27098727 PMC4839080

[ref4] Brown E. M. Clardy J. Xavier R. J. (2023). Gut microbiome lipid metabolism and its impact on host physiology. Cell Host Microbe 31, 173–186. doi: 10.1016/j.chom.2023.01.009, PMID: 36758518 PMC10124142

[ref5] De Bandt J. P. Waligora-Dupriet A. J. Butel M. J. (2011). Intestinal microbiota in inflammation and insulin resistance: relevance to humans. Curr. Opin. Clin. Nutr. Metab. Care 14, 334–340. doi: 10.1097/mco.0b013e328347924a, PMID: 21587065

[ref6] El Kaoutari A. Armougom F. Gordon J. I. Raoult D. Henrissat B. (2013). The abundance and variety of carbohydrate-active enzymes in the human gut microbiota. Nat. Rev. Microbiol. 11, 497–504. doi: 10.1038/nrmicro3050, PMID: 23748339

[ref7] Fan Y. Pedersen O. (2021). Gut microbiota in human metabolic health and disease. Nat. Rev. Microbiol. 19, 55–71. doi: 10.1038/s41579-020-0433-9, PMID: 32887946

[ref8] Forslund K. Hildebrand F. Nielsen T. Falony G. Le Chatelier E. Sunagawa S.. (2015). Disentangling type 2 diabetes and metformin treatment signatures in the human gut microbiota. Nature 528, 262–266. doi: 10.1038/nature15766 26633628 PMC4681099

[ref9] Gao Y. Zhao T. Lv N. Liu S. Yuan T. Fu Y.. (2024). Metformin-induced changes of the gut microbiota in patients with type 2 diabetes mellitus: results from a prospective cohort study. Endocrine 85, 1178–1192. doi: 10.1007/s12020-024-03828-x, PMID: 38761345

[ref10] Hang L. Wang E. Feng Y. Zhou Y. Meng Y. Jiang F.. (2022). Metagenomics and metabolomics analysis to investigate the effect of Shugan decoction on intestinal microbiota in irritable bowel syndrome rats. Front. Microbiol. 13:1024822. doi: 10.3389/fmicb.2022.1024822, PMID: 36478867 PMC9719954

[ref11] Heng X. P. Wang Z. T. Li L. Yang L. Q. Huang S. P. (2022). Mechanisms of Dangua recipe in improving glycolipid metabolic disorders based on transcriptomics. Chin. J. Integr. Med. 28, 130–137. doi: 10.1007/s11655-021-3337-2, PMID: 34755288

[ref12] Heng X. P. Wang Z. T. Li L. Yang L. Q. Huang S. P. Wang X. L.. (2023). Study on the regulation of miR-34a/Nampt axis in diabetic rats by Dangua fang. Chin. J. Integr. Med. 43, 184–192.

[ref13] Heng X. P. Yang L. Q. Huang S. P. Li L. Pan X. D. Lin Q.. (2019). Clinical study on the intervention of Dangua Humai oral liquid in major cardiovascular risk factors of patients with type 2 diabetes. Chin. J. Integr. Med. 39, 275–281.

[ref14] Human Microbiome Project Consortium (2012). Structure, function and diversity of the healthy human microbiome. Nature 486, 207–214. doi: 10.1038/nature11234, PMID: 22699609 PMC3564958

[ref15] Johnson E. L. Heaver S. L. Walters W. A. Ley R. E. (2017). Microbiome and metabolic disease: revisiting the bacterial phylum Bacteroidetes. J. Mol. Med. (Berl) 95, 1–8. doi: 10.1007/s00109-016-1492-2, PMID: 27900395 PMC5187364

[ref16] Koh A. De Vadder F. Kovatcheva-Datchary P. Bäckhed F. (2016). From dietary fiber to host physiology: short-chain fatty acids as key bacterial metabolites. Cell 165, 1332–1345. doi: 10.1016/j.cell.2016.05.041, PMID: 27259147

[ref17] Kubo M. (2020). Diurnal rhythmicity programs of microbiota and transcriptional oscillation of circadian regulator, NFIL3. Front. Immunol. 11:552188. doi: 10.3389/fimmu.2020.552188, PMID: 33013924 PMC7511535

[ref18] Lin Y. N. Hsu J. R. Wang C. L. Huang Y. C. Wang J. Y. Wu C. Y. (2024). Nuclear factor interleukin 3 and metabolic dysfunction-associated fatty liver disease development. Commun Biol. 7:897. doi: 10.1038/s42003-024-06565-z, PMID: 39048678 PMC11269659

[ref19] Ma W. W. Huang Z. Q. Liu K. Li D. Z. Mo T. L. Liu Q. (2024). The role of intestinal microbiota and metabolites in intestinal inflammation. Microbiol. Res. 288:127838. doi: 10.1016/j.micres.2024.127838, PMID: 39153466

[ref20] Magne F. Gotteland M. Gauthier L. Zazueta A. Pesoa S. Navarrete P.. (2020). The Firmicutes/Bacteroidetes ratio: a relevant marker of gut dysbiosis in obese patients? Nutrients 12:1474. doi: 10.3390/nu12051474, PMID: 32438689 PMC7285218

[ref21] Matsumura T. Ohta Y. Taguchi A. Hiroshige S. Kajimura Y. Fukuda N.. (2021). Liver-specific dysregulation of clock-controlled output signal impairs energy metabolism in liver and muscle. Biochem. Biophys. Res. Commun. 534, 415–421. doi: 10.1016/j.bbrc.2020.11.066, PMID: 33256979

[ref22] Mukhopadhya I. Hansen R. El-Omar E. M. Hold G. L. (2012). IBD-what role do Proteobacteria play? Nat. Rev. Gastroenterol. Hepatol. 9, 219–230. doi: 10.1038/nrgastro.2012.14, PMID: 22349170

[ref23] Niu J. Sun Y. Chen B. Zheng B. Mino-Kenudson M. Frank D. A.. (2020). Retraction note: fatty acids and cancer-amplified ZDHHC19 promote STAT3 activation through S-palmitoylation. Nature 583:154. doi: 10.1038/s41586-020-2414-6, PMID: 32555452 PMC7366828

[ref24] Perler B. K. Friedman E. S. Wu G. D. (2023). The role of the gut microbiota in the relationship between diet and human health. Annu. Rev. Physiol. 85, 449–468. doi: 10.1146/annurev-physiol-031522-092054 36375468

[ref25] Qin J. Li Y. Cai Z. Li S. Zhu J. Zhang F.. (2012). A metagenome-wide association study of gut microbiota in type 2 diabetes. Nature 490, 55–60. doi: 10.1038/nature11450, PMID: 23023125

[ref26] Ramadass B. Rani B. S. Pugazhendhi S. John K. R. Ramakrishna B. S. (2017). Faecal microbiota of healthy adults in South India: comparison of a tribal & a rural population. Indian J. Med. Res. 145, 237–246. doi: 10.4103/ijmr.ijmr_639_14, PMID: 28639601 PMC5501057

[ref27] Rivière A. Selak M. Lantin D. Leroy F. De Vuyst L. (2016). Bifidobacteria and butyrate-producing colon bacteria: importance and strategies for their stimulation in the human gut. Front. Microbiol. 7:979. doi: 10.3389/fmicb.2016.00979, PMID: 27446020 PMC4923077

[ref28] Salles B. I. M. Cioffi D. Ferreira S. R. G. (2020). Probiotics supplementation and insulin resistance: a systematic review. Diabetol. Metab. Syndr. 12:98. doi: 10.1186/s13098-020-00603-6, PMID: 33292434 PMC7656736

[ref29] Schneider E. O'Riordan K. J. Clarke G. Cryan J. F. (2024). Feeding gut microbes to nourish the brain: unravelling the diet-microbiota-gut-brain axis. Nat. Metab. 6, 1454–1478. doi: 10.1038/s42255-024-01108-6, PMID: 39174768

[ref30] Shalon D. Culver R. N. Grembi J. A. Folz J. Treit P. V. Shi H.. (2023). Profiling the human intestinal environment under physiological conditions. Nature 617, 581–591. doi: 10.1038/s41586-023-05989-7, PMID: 37165188 PMC10191855

[ref31] Sun S. Zhang R. Chen Y. Xu Y. Li X. Liu C.. (2024). Correction: E4bp4-Cyp3a11 axis in high-fat diet-induced obese mice with weight fluctuation. Nutr. Metab. (Lond.) 21:51. doi: 10.1186/s12986-024-00810-2, PMID: 39030626 PMC11264893

[ref32] Sze M. A. Schloss P. D. (2016). Looking for a signal in the noise: revisiting obesity and the microbiome. MBio 7:e01018-16. doi: 10.1128/mbio.01018-16 27555308 PMC4999546

[ref33] Tang X. Yang L. Miao Y. Ha W. Li Z. Mi D. (2023). Angelica polysaccharides relieve blood glucose levels in diabetic KKAy mice possibly by modulating gut microbiota: an integrated gut microbiota and metabolism analysis. BMC Microbiol. 23:281. doi: 10.1186/s12866-023-03029-y, PMID: 37784018 PMC10546737

[ref34] Tett A. Huang K. D. Asnicar F. Fehlner-Peach H. Pasolli E. Karcher N.. (2019). The *Prevotella copri* complex comprises four distinct clades underrepresented in westernized populations. Cell Host Microbe 26, 666–679.e7. doi: 10.1016/j.chom.2019.08.018, PMID: 31607556 PMC6854460

[ref35] Tsai Y. L. Lin T. L. Chang C. J. Wu T. R. Lai W. F. Lu C. C.. (2019). Probiotics, prebiotics and amelioration of diseases. J. Biomed. Sci. 26:3. doi: 10.1186/s12929-018-0493-6, PMID: 30609922 PMC6320572

[ref36] Ullah H. Arbab S. Tian Y. Chen Y. Liu C. Li Q.. (2024). Crosstalk between gut microbiota and host immune system and its response to traumatic injury. Front. Immunol. 15:1413485. doi: 10.3389/fimmu.2024.1413485, PMID: 39144142 PMC11321976

[ref37] Walters W. A. Xu Z. Knight R. (2014). Meta-analyses of human gut microbes associated with obesity and IBD. FEBS Lett. 588, 4223–4233. doi: 10.1016/j.febslet.2014.09.039, PMID: 25307765 PMC5050012

[ref38] Wang Y. Kuang Z. Yu X. Ruhn K. A. Kubo M. Hooper L. V. (2017). The intestinal microbiota regulates body composition through NFIL3 and the circadian clock. Science 357, 912–916. doi: 10.1126/science.aan0677, PMID: 28860383 PMC5702268

[ref39] Wang T. Wang R. X. Colgan S. P. (2024). Physiologic hypoxia in the intestinal mucosa: a central role for short-chain fatty acids. Am. J. Physiol. Cell Physiol. 327, C1087–C1093. doi: 10.1152/ajpcell.00472.2024, PMID: 39159391 PMC11482044

[ref40] Wang S. Yang M. Li P. Wong A. Rodrigues K. Lank D.. (2023). High-fat diet-induced DeSUMOylation of E4BP4 promotes lipid droplet biogenesis and liver steatosis in mice. Diabetes 72, 348–361. doi: 10.2337/db22-0332, PMID: 36508222 PMC9935497

[ref41] Wit M. Trujillo-Viera J. Strohmeyer A. Klingenspor M. Hankir M. Sumara G. (2022). When fat meets the gut-focus on intestinal lipid handling in metabolic health and disease. EMBO Mol. Med. 14:e14742. doi: 10.15252/emmm.202114742, PMID: 35437952 PMC9081902

[ref42] Wu X. Xie H. Yu G. Hebert T. Goh B. C. Smith S. R.. (2009). Expression profile of mRNAs encoding core circadian regulatory proteins in human subcutaneous adipose tissue: correlation with age and body mass index. Int. J. Obes. 33, 971–977. doi: 10.1038/ijo.2009.137, PMID: 19597517 PMC2743775

[ref43] Wu X. Y. Zheng X. M. Ye G. (2024). WGCNA combined with machine learning to explore potential biomarkers and treatment strategies for acute liver failure, with experimental validation. iLIVER 3:100133. doi: 10.1016/j.iliver.2024.100133 PMC12212670 40635857

[ref44] Xianpei H. Zhita W. Liang L. I. Liuqing Y. Suping H. Lang J.. (2024). Mechanisms of Dangua fang in multi-target and multi-method regulation of glycolipid metabolism based on phosphoproteomics. J. Tradit. Chin. Med. 44, 334–344. doi: 10.19852/j.cnki.jtcm.20230908.001, PMID: 38504539 PMC10927395

[ref45] Xianpei H. Zhita W. Liuqing Y. Liang L. I. Suping H. (2023). Dangua fang regulating tricarboxylic acid cycle and respiratory chain and its mechanism in diabetic rats. J. Tradit. Chin. Med. 43, 1150–1159. doi: 10.19852/j.cnki.jtcm.20230904.002, PMID: 37946477 PMC10623262

[ref46] Yang M. Zhang D. Zhao Z. Sit J. Saint-Sume M. Shabandri O.. (2020). Hepatic E4BP4 induction promotes lipid accumulation by suppressing AMPK signaling in response to chemical or diet-induced ER stress. FASEB J. 34, 13533–13547. doi: 10.1096/fj.201903292rr, PMID: 32780887 PMC8011030

[ref47] Yatsunenko T. Rey F. E. Manary M. J. Trehan I. Contreras M. Magris M.. (2012). Human gut microbiome viewed across age and geography. Nature 486, 222–227. doi: 10.1038/nature11053, PMID: 22699611 PMC3376388

[ref48] Zhang M. Feng R. Yang M. Qian C. Wang Z. Liu W.. (2019). Effects of metformin, acarbose, and sitagliptin monotherapy on gut microbiota in Zucker diabetic fatty rats. BMJ Open Diabetes Res. Care 7:e000717. doi: 10.1136/bmjdrc-2019-000717, PMID: 31641523 PMC6777410

[ref49] Zhang K. Yang C. Zhou X. Liang J. Guo J. Li M.. (2023). TRIM21 ameliorates hepatic glucose and lipid metabolic disorders in type 2 diabetes mellitus by ubiquitination of PEPCK1 and FASN. Cell. Mol. Life Sci. 80:168. doi: 10.1007/s00018-023-04820-w, PMID: 37249651 PMC10229743

[ref50] Zhao Z. Yin L. Wu F. Tong X. (2021). Hepatic metabolic regulation by nuclear factor E4BP4. J. Mol. Endocrinol. 66, R15–R21. doi: 10.1530/jme-20-0239, PMID: 33434146 PMC7808567

[ref51] Zou Y. Ju X. Chen W. Yuan J. Wang Z. (2020). Rice bran attenuated obesity via alleviating dyslipidemia, browning of white adipocytes and modulating gut microbiota in high-fat diet-induced obese mice. Food Funct. 11, 2406–2417. doi: 10.1039/c9fo01524h 32129359

